# Neuroglobin and Cytoglobin in Mammalian Nervous Systems: About Distribution, Regulation, Function, and Some Open Questions

**DOI:** 10.3390/brainsci15080784

**Published:** 2025-07-23

**Authors:** Stefan Reuss

**Affiliations:** Department of Nuclear Medicine, University Medical Center, Johannes Gutenberg-University, 55131 Mainz, Germany; reuss@uni-mainz.de

**Keywords:** cytoglobin, neuroglobin, expression pattern, CNS, PNS, nitric oxide

## Abstract

Globins are a class of globular proteins that function in the transportation or storage of oxygen. They are critical for cellular metabolism. Notable examples include hemoglobin, which is found in red blood cells, and myoglobin, which is present in muscle cells. Approximately two decades ago, a third globin, designated as neuroglobin, was identified, expressed predominantly in neuronal cells. This was followed two years later by the fourth, cytoglobin, found in cells of the fibroblast lineage, as well as in neuronal cell populations of the central and nervous systems. Both neuroglobin and cytoglobin have been found in the sensory and endocrine systems, albeit inconsistently, and it is thought that they are engaged in functions such as oxygen transport and storage, scavenging of free radicals, NO metabolism, peroxidase activity, and signaling functions. Neuroglobin is also expressed in astrocytes under challenging conditions. Common neuroscience methods were utilized to study the distribution and regulation of globin tissues and of single brain cells. Despite considerable overlap in the findings of various studies, some results deviate significantly from other studies. The potential causes of these discrepancies may include variations in detection methods, animal age and sex, time of day and year, and differing cell culture conditions. This review will explore factors that may influence functional aspects of globins and their detection in the mammalian brain.

## 1. Introduction

The oxygen-binding and -transporting proteins hemoglobin and myoglobin in mammals are well known and have been extensively investigated in a plethora of studies since their initial descriptions in 1878 and 1945, respectively.

Burmester and colleagues [1] were the first to document the presence of a third globin type, designated as neuroglobin (Ngb), within the nervous tissues of humans and mice. This discovery initiated a comprehensive research initiative focused on the identification of additional globins and the subsequent investigation of the distribution, regulation, and function of Ngb. The protein is expressed predominantly in the cytoplasm of nervous and endocrine cells and is able to bind gaseous ligands such as O_2_, NO, and CO. It also displays (pseudo)enzymatic properties (e.g., O_2_-mediated detoxification of NO, a molecule involved in intracellular and intercellular signaling) (cf. [2]). It is a monomer of approximately 150 amino acids in length, with a molecular mass of ~16 kDa and a P50 value for O_2_ binding in the range of 2–10 Torr depending on pH, temperature, and redox state. A number of comprehensive reviews covered aspects of the expression and putative functional roles of Ngb (for example, [3,4,5,6,7,8,9,10,11,12,13,14,15]).

Shortly thereafter a fourth globin, namely cytoglobin, was discovered independently by three groups [16,17,18] as a dimer of approximately 21 kDa subunits with an O_2_ affinity in the myoglobin-like range of ≈1 Torr. Initially, it was also named histoglobin or stellate cell activation-associated protein (STAP), given that the protein and its mRNA were found to be upregulated during hepatic cell activation by oxidative stress [19]. However, the name cytoglobin (Cygb) has become established. Its expression, in contrast to Ngb, was detected in the cytoplasm of non-neuronal cells of the fibroblast lineage (e.g., connective tissue, chondroblasts, osteoblasts, hepatic stellate cells), as well as in some neurons of the central nervous system (CNS) and peripheral nervous system (PNS), where the protein is also found in the cell nucleus. Comprehensive reviews of the literature pertaining to the expression, regulation, and potential functions of Cygb can be found in the following references: (for example, [6,8,9,10,12,13,20,21]). Moreover, a number of functions, including tumor suppression, have been demonstrated or hypothesized in non-neural tissues, particularly in the case of Cygb (see [20] for a comprehensive review).

Subsequent years have seen the discovery of additional globin types, including globin E, globin X, globin Y, and androglobin (cf. [6,9]). However, these will not be covered in this study, as they are not expressed in neural tissues and/or are not found in mammals.

Presently, the search for expression parameters, regulatory factors, and functional aspects of Ngb and Cygb is ongoing. In addition to the generally accepted functions of these proteins, such as oxygen transport and storage, as well as the detoxification of excess nitric oxide, non-canonical functions have been identified. However, numerous research questions remain unanswered, and addressing these issues could significantly contribute to our understanding of the functional characteristics of these globins.

As has been observed in general (cf., for example, [22,23]), globin research is also affected by issues of reproducibility and replication in science. This may be attributable, at least in part, to the utilization of disparate methods and the presence of variations and inaccuracies in critical parameters such as the application and blocking of primary antibodies, fixation, incubation, analyzer blinding, and the inadequacy of the researcher’s expertise in brain science. Furthermore, a number of physiological factors that possibly were not taken into account or are still unidentified may blur the findings. The presence of mixed findings underscores the necessity for further investigation. The present paper aims to review consistencies and discrepancies, as well as to offer some possible reasons for diverging results. This analysis will be conducted from the perspective of a systems neuroscientist, as opposed to a molecular biology approach. The distribution of Ngb and Cygb in the CNS, PNS, and associated structures will be summarized, and potential reasons for variability in results will be highlighted. However, it should be noted that no significant differences were observed between related species such as mice and rats when the same method was used. Basic observations were also confirmed in individual cases on human post-mortem material.

The review will also encompass aspects not previously addressed, including anatomical distribution, methodical elements that may influence detection or expression, and potential roles in functional systems.

## 2. Distribution of Neuroglobin and Cytoglobin in the CNS

The majority of globin expression sites were found in the nervous system. The CNS, and, in particular, the brain, has been the focus of extensive research. The subsequent summary will provide a concise overview of the current understanding of both globins in neural tissues.

### 2.1. Neuroglobin

Burmester and colleagues [1] were the first to describe the expression of Ngb-mRNA and protein at different levels in many brain regions of mice and humans. Northern blots revealed strong signals in regions exhibiting high neuronal density and/or large cell somata, including the cerebral cortex, olfactory bulb, thalamus, and pontine nuclei. These findings were corroborated by in situ hybridization (ISH) and immunohistochemistry (IHC) and further expanded upon through numerous brain regions and enhanced scanning methods. This observation comprises the hippocampal formation, as well as the hypothalamic and septal nuclei. The studies thus far have encompassed the analysis of human, rat, mouse, and gerbil brains.

A more or less global expression of Ngb-mRNA was observed by in situ hybridization (ISH) [1,24,25]. While in the study by Mammen and colleagues [25], focal concentrations in mice diencephalic aspects, particularly in medially located hypothalamic regions, were demonstrated. The figures also provide evidence for additional weak ubiquitous expression. Conversely, Ngb expressed diffusely throughout the brain was suggested by IHC from several mouse brain regions [26]. In rats, Ngb-mRNA and protein were found in cortical, amygdaloid, hypothalamic, and tegmental nuclei, but it should be emphasized that this relates to regional rather than single-cell co-expression.

Immunohistochemistry indicated a relatively distinct expression of Ngb protein [27,28,29,30,31,32]. Preferential expression was observed in, e.g., the hypothalamus [27,28] and the auditory brainstem [33]. Analogous patterns were observed in the human brain [33,34]. Parenthetically, the results of these authors should be interpreted with some restraint since their Ngb antibody-labeled neuronal nuclei was not found in other studies and was also not apparent in our material [30,32,33].

**Figure 1:**
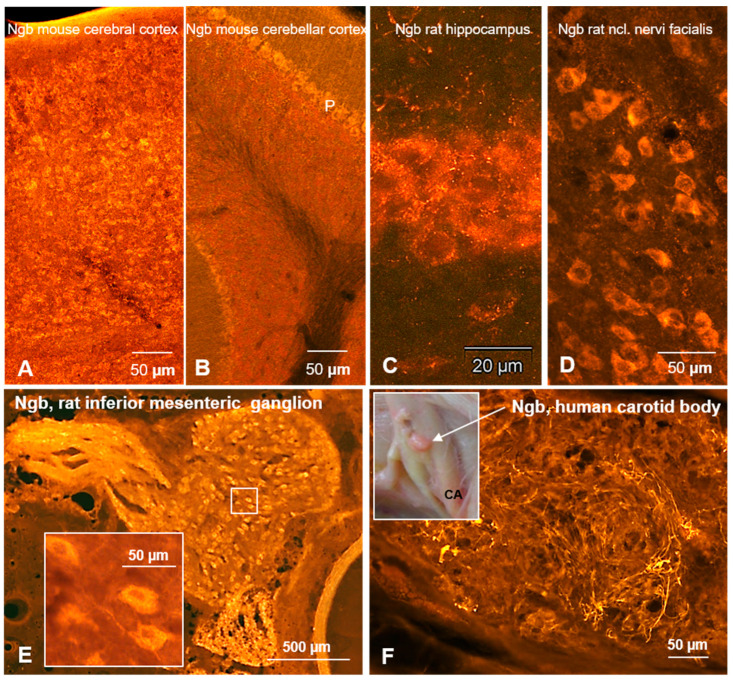
Neuroglobin in mammalian CNS and PNS. (**A**–**D**) Frontal sections. (**A**) Neurons are immunolabeled in all layers of the cerebral cortex. (**B**) In the cerebellar cortex, some small neurons and particularly large Purkinje (P) cells and their processes exhibit immunofluorescence. (**C**) In the hippocampus, large Ngb-immunoreactive pyramidal neurons are present. In brainstem nuclei such as the nucleus of the facial nerve (**D**), many, if not all, neurons express neuroglobin. (**E**) Ngb-immunoreactive neurons in the rat inferior mesenteric ganglion, shown in the insert in higher magnification. Note that neuronal perikarya and processes are labeled but nuclei are not (in contrast to cytoglobin). (**F**) Human glomus caroticum (carotid body), located in the bifurcation of a common carotid artery (CA) insert. The magnified section depicts neuroglobin-immunoreactive nerve fibers, likely stemming from the glossopharyngeal nerve.

**Figure 2:**
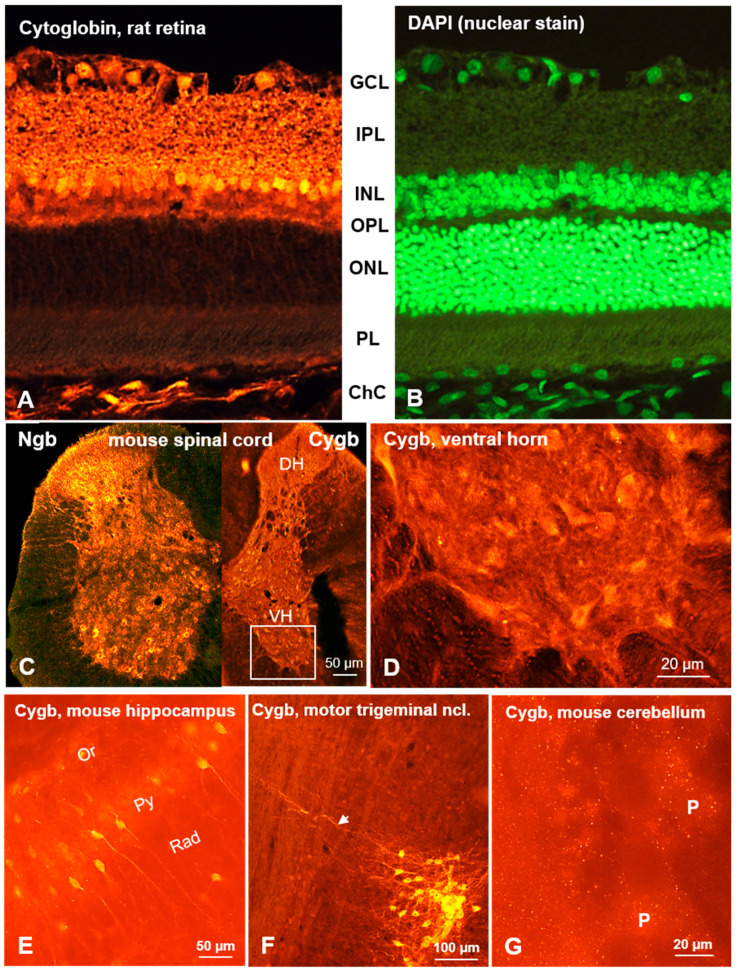
Cytoglobin immunofluorescence in rodent neuronal tissues. (**A**) Rat retina demonstrating Cygb expression in neuronal somata of the ganglion cell layer (GCL) and inner nucleus layer (INL); dense Cygb puncta in the inner plexiform layer (IPL) and outer plexiform layer (OPL), as well as in the unlabeled outer nuclear layer (ONL) and photoreceptor layer (PL). Note the strong labeling of fibroblasts in the choroid coat (ChC, vascular layer). (**B**) Cell nuclei stained by DAPI, same section. (**C**,**D**) Mouse spinal cord, showing Cygb neurons predominantly in the central gray and ventral horn, from which large Cygb-labeled motoneurons are shown under higher magnification (**D**). Note the different expression of Ngb as shown on the left side of the spinal cord. (**E**) The medial CA1 region of the hippocampus demonstrates strong immunostaining of neurons in the pyramidal cell layer (Py); a few neurons from the oriens layer (Or) and stratum radiatum (Rad) were also stained. (**F**) Strong staining of neurons in the motor trigeminal nucleus. (**G**) Higher magnification demonstrating dense IR puncta surrounding unlabeled Purkinje cell somata (P) of the ganglion cell layer. Note the long processes of pyramidal cells in (**E**) and trigeminal cells in (**F**) labeled by the Cygb antibody, as well as the puncta surrounding (unlabeled) Purkinje cells (P) in (**G**).

Ngb neurons have also been demonstrated in the rat hippocampus (CA3, dentate gyrus; Figure 1C), medial habenular nucleus, laterodorsal thalamus, and retrosplenial granular cortex [12,27,35]. Figure 1 exemplarily demonstrates Ngb immunofluorescent neurons in the cerebral cortex (Figure 1A), cerebellar cortex (Figure 1B), hippocampus (Figure 1C), and the nucleus of the facial nerve (Figure 1D). Only a single paper described cellular Ngb distribution in the spinal cord, where large motoneurons in the ventral horn were labeled [12]. In addition, primary afferent Ngb fibers originating from the dorsal root ganglion [13] are present in the dorsal horn, which was interestingly not seen in Cygb immunostaining (Figure 2C,D). This fits perfectly with the finding that neurons of the spinal ganglion are immunoreactive to Ngb but not to Cygb.

It is noteworthy that the low expression levels observed in the cerebellum and spinal cord are particularly salient when considering the minimal representation of these regions within the tissue probe. However, it is crucial to recognize that the absence of a strong signal in a particular tissue probe does not preclude the possibility of a robust signal in a distinct cell type, as evidenced by the significant expression observed in cerebellar Purkinje cells and spinal motor neurons.

With regard to the intracellular location of Ngb, the majority of molecules were detected in the cytosol, while a small proportion of Ngb proteins were localized to the mitochondria [36]. The study also revealed that the level of mitochondrial Ngb increased after oxygen–glucose deprivation in mouse cortical neurons in vitro, suggesting translocation under challenging conditions [36].

Notably, Ngb has also been observed in astrocytes under physiological conditions involving augmented oxygen demand, such as in deep-diving [31,37], in subterranean living animals [38], in cell culture [39], or in neuropathological models [40,41]. For a more in-depth examination of this topic, please refer to Section 6.

### 2.2. Cytoglobin

The fourth globin detected, cytoglobin (Cygb) [16,17,18], was identified in neurons throughout the brain in rats, mice, and humans, which are the only mammalian species that have hitherto been studied in this context.

Northern blot analysis [16] revealed the presence of Cygb signal in various brain regions, including the amygdala, caudate nucleus, cerebellum, cerebral cortex, frontal lobe, hippocampus, medulla oblongata, putamen, substantia nigra, temporal lobe, thalamus, subthalamic nucleus, and spinal cord. It should be noted parenthetically that Cygb from pericytes, contractile cells attached to the exterior of capillaries, may be a constituent of the Cygb signal in blots from brain homogenates (see Section 12.2 for further details).

Immunohistochemical studies on brain sections demonstrated that these signals stem from neurons that exhibit a differential distribution, with dense clusters in some regions and loose dissemination in others. The distribution in a cross section from the mouse spinal cord, as demonstrated by IHC, is demonstrated in Figure 2C,D. Cygb neurons with large processes were seen in brain regions such as the hippocampus (Figure 2E) and the motor trigeminal nucleus (Figure 2F). In the cerebellum (Figure 2G), Purkinje cells were unlabeled—in contrast to Ngb expression (Figure 1B)—but immunoreactive puncta likely representing presynaptic structures were clearly seen (Figure 2G). It is conceivable that they belong to a large number of climbing fiber synapses originating from the inferior olivary nucleus that provide strong excitatory input to the Purkinje cell soma.

However, initial IHC studies investigating specific brain regions revealed the presence of Cygb neurons in several locations [42,43,44]. The application of quantitative methods yielded congruent results regarding Cygb expression in the mouse brain [45]. However, a detailed demonstration of the location of Cygb neurons is available only for mice [27,46]. A consensus emerges from these studies, highlighting a widespread distribution of the protein within a limited number of cells. Interestingly, the study by Hundahl and coworkers [27] also demonstrated a clear, but not complete, regional coincidence of Ngb and Cygb proteins, as well as of mRNA and proteins.

In a given neuronal cell, Cygb immunoreaction has been observed in the cell soma (i.e., nucleus and cytoplasm) and in processes (i.e., axon and dendrites). The finding by IHC of nuclear Cygb is in accordance with the results of other studies, and has been confirmed through a combination of methods such as cell fractioning and Western blot analysis [47], as well as living cell imaging of cytoglobin fused to the nuclear localization factor [48]. However, the mechanism of Cygb translocation from cytoplasm into the nucleus remains to be determined. Nuclear transfection assays with green fluorescent protein fusion constructs have demonstrated the absence of active nuclear import [42,49], but suggest the presence of a neuron-specific factor that facilitates the transport or diffusion of the protein from the cytoplasm to the nucleus. In fact, molecules with a molecular weight less than 40 kDa have been found to be capable of passively equilibrating via nuclear pore complexes [50]. The localization of these molecules is consistent with a potential role of cytoglobin in protecting genomic DNA from oxidant-induced damage during ischemic injury.

It is important to note that, in contrast to neurons, non-neuronal cells of the fibroblast lineage did not demonstrate nuclear staining, despite the presence of clear Cygb expression in the cytoplasm.

A second feature that is clearly different between Ngb and Cygb expression is the expression in glial cells. While Ngb protein was observed in astrocytes under physiological conditions of augmented oxygen demand (see Section 6), there was no evidence that Cygb is expressed in glia under any conditions.

## 3. Distribution of Neuroglobin and Cytoglobin in the PNS

Less information is available on the distribution of both globins in the PNS, as only few studies provide globin-related descriptions of peripheral ganglia and nerve pathways.

### 3.1. Neuroglobin

In the rat dorsal root ganglion, apparently all neuronal perikarya and axons were labeled [13]. These axons provide primary afferent structures to recipient laminae in the dorsal horn of the spinal cord, which were also labeled (Figure 2C). Furthermore, immunoreactive perikarya and processes (putatively axons) are present in the rat inferior mesenteric ganglion (Figure 1E) and were previously observed in the inner ear spiral ganglion of rats, mice, and humans [33,51,52]. Additional ganglia were, to my knowledge, not studied.

### 3.2. Cytoglobin

There is currently no evidence that cells in peripheral ganglia such as the sensory trigeminal and spinal ganglia, as well as the sympathetic superior cervical ganglion, express Cygb [46]. In the latter, only a few fibers of passage were found to be Cygb-immunoreactive. It is notable that other publications addressing Cygb expression make no mention of ganglia.

### 3.3. Carotid Body (CB; Glomus Caroticum)

With regard to the presence of globins in other peripheral structures, information concerning this important oxygen-sensing structure [53] is missing. For both Ngb and Cygb, compelling evidence for respective immunoreactivity in the CB was not presented.

Our own study showed distinct Ngb-labeled structures that appeared to be large processes or fibers extending over many CB regions (Figure 1F). These most likely represent axons of the glossopharyngeal nerve, i.e., afferent axons to glomus type I cells, which represent chemoreceptors detecting predominantly the partial pressure of arterial oxygen (cf. [54]). These cells, as well as the glia-like supporting sustentacular or glomus type II cells, are not known to be equipped with large processes. The subject of globin transport in fibers will be addressed in the next section.

## 4. Globins in Nerve Fibers?

Nerve fibers are composed of axons and supporting glial cells such as oligodendrocytes in the CNS and Schwann cells in the PNS. It can be assumed that the presence of globin staining in nerve fibers indicates the presence of nerve cell axons that express globin.

Overall, there is only anecdotal evidence for globins in nerve fibers, while systematic studies on this phenomenon are lacking. In addition, the significance of this staining, whether it pertains to synthesis, transport, or storage mechanisms of the protein, remains to be elucidated.

Neuroglobin-stained fiber bundles have been observed in the Corpus callosum, which connects the brain hemispheres, and in the white matter of the spinal cord, consisting of bundles of axons that transmit nerve signals up and down the spinal cord [12], as well as in primary afferent fibers in the dorsal horn (Figure 2C). The axonal presence of mitochondria, but not ribosomes, serves to reinforce the hypothesis of an association between Ngb and mitochondria.

The fasciculus retroflexus, which connects the interpeduncular with the medial habenular nucleus, exhibited Ngb staining in mice [27]. Furthermore, the cochlear nerve, comprising the axons of Ngb-containing spiral ganglion cells [33], and nerve fibers in the carotid body (Figure 1F) of putative glossopharyngeal origin, are Ngb-positive. Other structures, such as the diagonal band of Broca, were Ngb-negative [27].

Punctate structures labeled by Cygb-ISH, which most probably represent presynaptic sites of Cygb synthesis, were observed in, e.g., the optic and olfactory nuclei [45]. Mitochondria, which globins are associated with, are transported by axonal microtubules and reach synaptic endbulbs. Additionally, these structures have been identified in dendrites, where they manifest as elongated forms in distal regions. Prior IHC studies have demonstrated neuroanatomical evidence for axonal Cygb transport over relatively long distances. This evidence includes the intense fiber staining observed in the fasciculus retroflexus (Meynert) and in the olfactory and optic nerves [46], but not in the diagonal band of Broca [27]. Although this question was not specifically addressed, it seems that globins are present predominantly in afferent and efferent nerve fibers, and not in commissural fibers.

## 5. Distribution of Neuroglobin and Cytoglobin in Sensory Systems

Immunoreactivities against Ngb or Cygb have been detected in peripheral sensory structures—with the exception of gustatory and skin sensory systems—and in corresponding downstream brain regions. The extant literature is focused predominantly on the visual and auditory systems, which are responsible for processing the highest data volumes at the fastest speeds. These senses, considered the most significant in humans and in many other mammals, demand the highest metabolic rates. The functional anatomy of globins in sensory systems will be discussed in greater detail in the following sections.

### 5.1. Visual System

#### 5.1.1. Neuroglobin

Of the sensory systems, the expression of Ngb has been most thoroughly studied in the visual pathway, particularly in the retina. The presence of the protein in the retina, which is one of the tissues in the body with the highest oxygen consumption, was predictable due to the structure’s role as a component of the CNS, originating as an outgrowth of the embryonic diencephalon. Despite the presence of some discrepancies between studies, the fundamental findings are largely consistent.

Ngb-mRNA has been detected in the nuclear and ganglionic layers, where the neuronal somata are located, while the protein has been found predominantly in the plexiform layers and photoreceptor inner segments, where synapses are the prevailing structures in both rodents and humans [43,55,56]. The synaptic layers stand out due to the location of the mitochondria, which consumes the most oxygen. In advanced glaucoma, a pathological situation in which the retina is under ischemic stress, increased expression was found to occur in these areas and in the nuclear layers [56]. Notably, experimental elevation of intraocular pressure, a major risk factor for glaucoma, resulted in a rapid and transient increase in retinal Ngb, as analyzed by Western blot [57].

In a separate study, the expression of Ngb protein was observed in various layers of the canine and human retinae, with the exception of the photoreceptor outer segments but including the pigment epithelium [58,59]. Conversely, other researchers identified Ngb-positive neuronal somata exclusively in the ganglion and inner nuclear layers of mouse retina [60]. Mathematical modeling has suggested that Ngb may enhance oxygen uptake by photoreceptor inner segments and inner plexiform layers, which are most susceptible to hypoxia [61]. A recent review of the expression of Ngb-mRNA or protein in different retinal cell types and its modulation after retinal injury was provided by Solar Fernandez et al. [62].

Downstream centers of visual processing exhibit distinct expressions of Ngb-mRNA and/or protein in neurons. These sites include the lateral geniculate nucleus, the striate and peristriate cortices, the superior colliculus, the pretectal area, and the tegmental terminal nucleus of the optic tract [24,27,32,63]. Furthermore, additional retinal afferents conveyed by ganglion cell axons are known to target the hypothalamic suprachiasmatic, paraventricular, and supraoptic nuclei, as well as dorsal raphe nuclei. These regions have been observed to contain Ngb-positive neurons.

The presence of certain inaccuracies in the methodical parameters makes the evaluation of the divergent findings a highly challenging task. For instance, retinal metabolism is contingent on both circadian clock adaptation and intrinsic light perception, suggesting that discrepancies in findings may be attributable to variations in ambient lighting conditions. Furthermore, the timing of the killing of animals and the harvesting of tissues has the potential to influence the outcomes.

The absence of details regarding fixation, post-mortem delays, and other parameters specific to human eyes poses a significant challenge in determining the validity of the findings. In addition to the limited number of specimens in studies such as of the post-mortem human eye, a number of other factors may influence the results (see Section 8 for further discussion).

**Figure 4:**
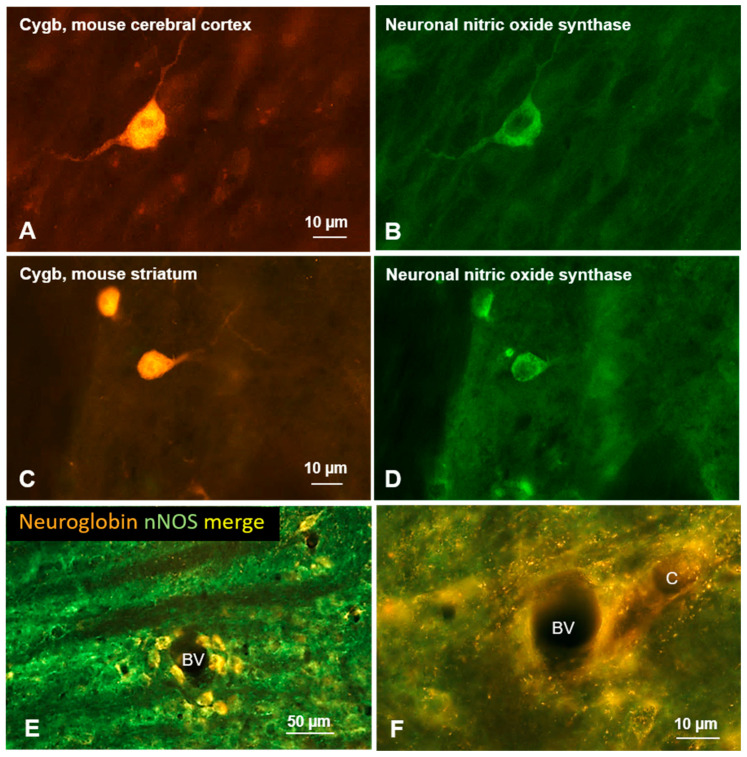
(**A**–**D**) Double immunofluorescent labeling of cytoglobin (left row) and neuronal nitric oxide synthase (right row) in frontal sections of the mouse brain. Complete or partial colocalization of both proteins was observed in several brain regions such as the cerebral cortex (**A**,**B**), and the striatum (**C**,**D**). Note that Cygb is present in the cytoplasm and cell nucleus, while nNOS is detected only in the cytoplasm. (**E**,**F**) Neurons labeled for neuroglobin (red) and for neuronal nitric oxide synthase (nNOS, green) in the rat auditory brainstem; (**E**) note that Ngb-immunoreactive neurons are concentrated within the vicinity of small blood vessels; (**F**) fine neuronal processes in apparent close contact to capillaries.

Ngb and/or further oxygen-binding proteins may contribute to “the opto-respiratory compromise: they must optimize oxygen delivery to the retina without blocking vision with blood” (cf. [64]). Consequently, Ngb may facilitate oxygen supply in retinae with diminished capillary density [65], thereby enhancing visual acuity (Ngb neurons in close vicinity to small blood vessels and capillaries in the brainstem are shown in Figure 4E,F). Indeed, differences in retinal layers with regard to Ngb expression between vascular (rat) and avascular (guinea pig) retinae were observed [65].

Given the paucity of knowledge surrounding the function of Ngb in the visual system in vivo, a study was conducted to measure oxygen fluxes in the retina of Ngb-KO animals. The non-invasive optical technique of broadband near-infrared spectroscopy revealed a suppressed signal associated with hemodynamics and oxidative metabolism in the retinae of Ngb-KO mice [66]. However, further research is required to ascertain whether visual parameters such as acuity, color vision, night vision, or signal discrimination are influenced by the absence of Ngb.

#### 5.1.2. Cytoglobin

As was the case with Ngb, Cygb was observed in the canine retinal ganglion cell layer, inner nuclear layer, inner and outer plexiform layers, photoreceptor inner segments, and pigment epithelium [58]. A similar distribution was observed in the human retina, where Cygb is co-expressed with Ngb in ganglion cells [59]. Furthermore, the optic nerve, which is composed of axons originating from retinal ganglion cells, also exhibited Cygb-IR [46]. In the rat retina, Cygb was observed in ganglion cells and in neurons of the inner nuclear layer, while putative synaptic structures were seen in the inner and outer plexiform layers (Figure 2A,B).

Furthermore, Cygb was detected by means of IHC in neurons of retino-recipient downstream structures, including the hypothalamus, the lateral geniculate body, and the superior colliculus [27,46], which indicates that Cygb (like Ngb) plays an important role in the visual system. It should be noted, however, that single-globin-expressing cells in the system were not confirmed to process visual information.

### 5.2. Auditory System

#### 5.2.1. Neuroglobin

In a seminal study published two decades ago, Chen and Liu [67] established that hypoxia potentiates noise-induced hearing loss by inducing damage to the cellular energy generation system. At that time, the potential involvement or even presence of Ngb or Cygb in the auditory system had not been investigated. Initial studies later detected Ngb in rat spiral ganglion neurons (SPNs) of the cochlea in rats, at both the mRNA and protein level [51]. It colocalized with cytochrome c (Cytc), the marker of oxidative metabolism. A similar distribution was found by IHC in the post-mortem human cochlea, with a significant decrease in Ngb-IR in spiral ganglion neurons (SGNs) from specimens with inner ear pathologies [52].

A comprehensive study demonstrated a distinct distribution of Ngb-IR neurons in the peripheral and central auditory system of rats, mice, and men [33]. Utilizing quantitative real-time RT-PCR, ISH, IHC and Western blot techniques, it was ascertained that neuroglobin is expressed at elevated levels in the cochlea and auditory brainstem of mice and rats when compared to whole brain levels. For primary cochlear neurons, Ngb expression is limited to the subpopulation of type I SGN, which innervate inner hair cells, while the subpopulation of type II SGN, which innervate the outer hair cells that do not express Ngb. Furthermore, immunoreactivity was observed in the basilar membrane and stria vascularis, a structure of paramount importance to hearing. No Ngb immunostaining was found in hair cells, supporting cells, or Schwann cells surrounding type I SGN. Conversely, auditory brainstem centers exhibited substantial Ngb expression in their neuronal perikarya, specifically in the cochlear nuclei and the superior olivary complex (SOC). The latter is a group of interconnected nuclei that facilitates binaural hearing, regulates the cochlear amplifier mechanism, and protects against acoustic injury. For a more detailed discussion and references, see [33,68].

It is noteworthy that the majority of olivocochlear neurons that provide the efferent innervation of outer hair cells, as identified by neuronal tract tracing, were Ngb-immunoreactive [33,68]. Ngb in the SOC frequently colocalized with neuronal nitric oxide synthase (nNOS), the enzyme responsible for nitric oxide (NO) production. It is highly probable that Ngb colocalizes with nNOS in SGN, since all of these cells appear to express nNOS [69].

In addition to the aforementioned aspects of the ascending and efferent systems, Ngb immunofluorescence was present in perikarya and fibers within the descending auditory system, including the auditory cortex, medial geniculate body, inferior colliculus, and lemniscal nuclei [33]. A subsequent study of auditory functions in Ngb-KO mice revealed that sensitive hearing, the detection of acoustic signals in a noisy environment, and the recovery of hearing ability after experimental acoustic trauma are impaired in Ngb-KO mice [70]. The involvement of Ngb in auditory neurons in noise-protecting mechanisms was recently reinforced by the finding that Ngb was among eight genes that were upregulated in the basal turn after oxidative stress to the cochlea [71].

#### 5.2.2. Cytoglobin

The available literature on the expression and potential function of Cygb in the auditory system is limited. The presence of this globin in dorsal and ventral cochlear nuclei and in the SOC, but not in the inferior colliculi, had been previously described [45,46]. A more detailed study of the central auditory system was conducted by immunohistochemistry [68], in which it was observed that many neurons expressed Cygb protein. Furthermore, quantitative data were collected, which demonstrated that a relatively high percentage of neurons in a given region were Cygb-positive in both rats and mice. However, a detailed multi-methodical analysis of cochlear and brainstem Cygb, as conducted for Ngb [33], is still lacking. It is noteworthy that the demonstration of Cygb-IR in identified olivocochlear neurons suggests that efferent synapses in hair cells contain Cygb. A similar finding was reported for Ngb, thus suggesting that both globins may contribute to metabolic parameters or the regulation of the cochlea. However, the question remains unsolved as to whether these neurons also transport Cygb-mRNA and/or protein into the organ of Corti. While Cygb-mRNA has been detected in the cochlea [72], the precise origin of this molecule remains uncertain, namely whether it originates from OCN axons, SGNs, or cochlear fibroblasts and pericytes. The authors suggested a local source [72]. This may consist of SGN, given the widespread co-expression of nNOS and Cygb as well as the clear presence of nNOS in SGN [69]. The brainstem has also been proposed as a potential source, given the mounting evidence for axonal mRNA transport and translation, which facilitates rapid responses to physiological demands (cf. [73]).

Cygb immunofluorescence of differential intensity was also observed in brain structures principally concerned with auditory functions in rats and mice. These include the auditory cerebral cortex, the medial geniculate body, the inferior colliculus, the lateral lemniscal nuclei, the cochlear nucleus, and the SOC. This finding pertains to both immunopositive neuronal cell bodies and fiber systems (see below), as well as punctate structures that are likely to be synapses.

To date, the auditory system in Cygb-KO mice has not been studied, but we would shed more light on the putative role of this globin in auditory function.

### 5.3. Vestibular System

Despite the seminal importance of the equilibrium sense, there is a paucity of data regarding the presence of Ngb or Cygb in peripheral vestibular organs.

The effects of globin knockouts were not specifically tested, but a failure of the system would most probably result in a significant phenotype. Thuy and colleagues [74] reported multiple organ abnormalities in Cygb-KO mice and noted that aged KO animals exhibited severe balance impairments.

From downstream structures in the system, neurons of the medial vestibular nucleus of the rat and mouse brainstems express Ngb [24] and Cygb [27,45,46]. Cygb puncta, presumed to be terminals, were observed in the brainstem nucleus, which receives input from vestibular end organs and other sources [46].

### 5.4. Olfactory System

It is not known whether primary sensory cells express either globin. Ngb has been detected in olfactory bulb periglomerular cells [75], while mitral cells have only been observed in transgenic Ngb-overexpressing animals [26].

Cygb has been described in mouse mitral and granular layers of the olfactory bulb and in olfactory nuclei [27,45,46], as well as in the olfactory nerve [27,46].

Given the importance of olfaction as an orientation tool in many species, such as rodents and canines, further studies are necessary to investigate the distribution of globins in this pathway and the possible consequences of globin loss in this sensory system.

### 5.5. Endocrine Systems

**Figure 3:**
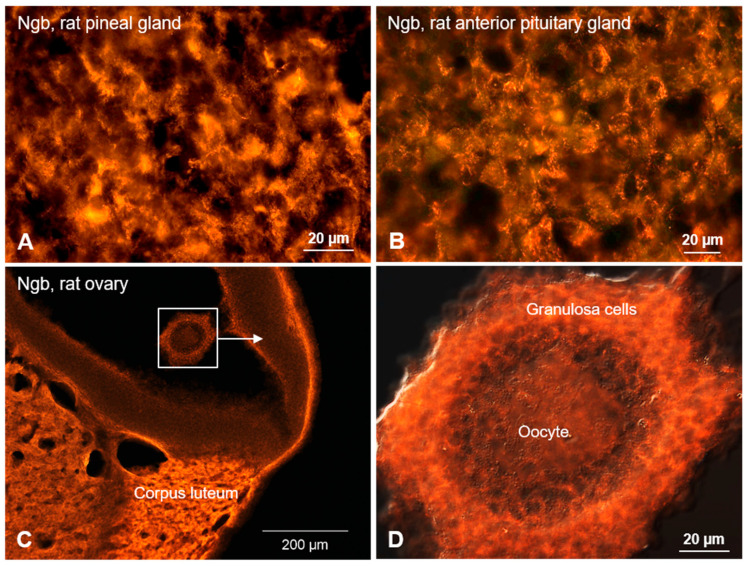
Ngb-immunofluorescence in rat endocrine cells. (**A**) Pinealocytes (parenchymal cells in the pineal gland producing predominantly serotonin and melatonin) express Ngb. (**B**) Similarly, cells of the anterior lobe of the pituitary gland (producing glandotrope hormones) are immunostained. (**C**) The rat ovary exhibits distinct staining of estrogen/progesterone-producing cells of the Corpus luteum. (**D**) Boxed area in C under higher magnification, featuring oocyte surrounded by granulosa cells (producing sex steroids).

The expression of Ngb-mRNA has been reported in several hormone-producing cell types of the testis, as well as of the pituitary and adrenal glands [1,24,76]. Figure 3 depicts endocrine cells expressing Ngb protein in the pineal gland (Figure 3A), anterior pituitary gland (Figure 3B) in the Corpus luteum of the rat ovary (Figure 3C), and in granulosa cells surrounding oocytes (Figure 3D).

Northern dot blot analysis of human tissues demonstrated the presence of Cygb-mRNA in the testis, ovary, pancreas, as well as the pituitary, adrenal, and thyroid glands [16]. However, the pineal gland, which was only studied in mice, was found to be negative for Cygb [46].

### 5.6. Cerebrospinal Fluid (CSF) and Blood

Given the established connection between the brain, blood, and CSF, which facilitates the exchange of substances, it was logical to investigate the presence of globins in these bodily fluids. Indeed, Ngb has been detected in the CSF of human subjects [77], as well as in blood serum, where its levels correspond to those observed in the cerebral cortex [35]. However, the precise mechanisms by which Ngb reaches CSF and serum remain to be elucidated. One hypothesis is that Ngb is secreted into blood and/or CSF. If so, by which cell type? It would be interesting to determine whether liquor-contact neurons in the vicinity of brain ventricles are involved in this exchange of Ngb. An interesting aspect in this regard is the flow of CSF through the perivascular space and the functional role of these cerebrovascular structures [78,79].

The presence of Cygb in CSF or serum remains to be elucidated. It is open whether this globin is not present in these body fluids or has not been studied in sufficient detail thus far.

## 6. Globin Expression in Glial Cells

Despite the absence of any documented evidence of Cygb expression in glial cells under any conditions within the mammalian nervous system, including the retina, several lines of evidence suggest that Ngb is expressed in glial cells in some situations.

It has been demonstrated that astrocytes express Ngb upon hypoxia. In deep-diving seals, no differences in total brain Ngb levels were observed in comparison to rodents; however, a different location of the globin was noted [31]. Ngb was expressed predominantly in astrocytes, which were identified by glial fibrillary acidic protein (GFAP) immunofluorescence, accompanied by a shift in the distribution of Cytc. A similar strategy to evade hypoxic insults was identified in the harp seal, while in the harbor porpoise and mink whale, Ngb expression remains restricted to neurons. The expression levels of Ngb, however, were found to be 4–15 times higher in porpoise and whale than in related terrestrial mammals and in seals [37].

Hypoxic situations for glial cells also include neuropathological events such as traumatic brain injury and autoimmune encephalitis. In appropriate in vivo mouse models, Ngb is expressed in reactive astrocytes [40]. The expression of Ngb in rat and human astrocytoma cell lines, as well as in astrocytoma tissue [80], suggests a mechanism by which tumor cells can to adapt to hypoxia. A similar means may underlie the expression of Ngb in primary cultures of astrocytes from a newborn mouse brain. Notably, Ngb antisense treatment has been observed to enhance apoptosis under these conditions [39].

With regard to the expression of Ngb in glial cells, it is noteworthy that astrocytes—the glial cell type found to express Ngb—constitute a mere one-fifth of these cells in the human brain, with oligodendrocytes accounting for 75%. The question of whether hypoxic states influence the metabolic behavior of oligodendrocytes and microglia requires further investigation. The prevailing interpretation of Ngb presence in astrocytes is that of protein synthesis; however, the possibility exists that, under specific conditions, neurons may produce excess Ngb, which is subsequently taken up by astrocytes, resulting in Ngb protein immunofluorescence in these cells. To the best of my knowledge, Ngb transcripts have only been detected on a single occasion in primary cultures of cortical astrocytes [39]. A comprehensive review provides further details on the role of Ngb in astrocytes and neurons for the mechanisms of neuroprotection [41].

## 7. Co-Expression of Globins with Neuroactive Substances

Few studies describe the phenotype of globin-expressing neurons in relation to colocalized macromolecules such as transmitters or enzymes. It appears that the co-expression with nNOS is the most common type for both globins.

### 7.1. Neuroglobin

In the rat brain, Ngb colocalizes with tyrosine hydroxylase in noradrenergic neurons and with choline acetyltransferase (ChAT) in tegmental nuclei. Co-expression with nNOS in tegmental, amygdaloid, and hypothalamic nuclei [29], as well as in the auditory brainstem [33], has also been described, albeit not completely concordant. Figure 4E,F demonstrates colocalization of Ngb and nNOS in cells neighboring small blood vessels or capillaries. Some of these cells may represent pericytes (see also Section 12).

Partial colocalization of Ngb with parvalbumin or ChAT was observed in mouse striatal interneurons, where all ChAT and parvalbumin neurons were also Ngb-positive [81]. In retinal ganglion cells, Ngb is partly co-expressed with Cygb [59].

### 7.2. Cytoglobin

The most frequently co-expressed molecule with Cygb was nNOS. Such a co-expression pattern was found in many mouse brain regions, such as the olfactory bulb, cerebral cortex (Figure 4A,B), striatum (Figure 4C,D), and vestibular nuclei [46]. However, quantification of double labeling is not available.

In the rat and human hippocampus, many Cygb neurons also co-expressed nNOS [44]. In rats, some hippocampal Cygb neurons also expressed parvalbumin or heme-oxygenase 1, while another distinct population expressed somatostatin and vasoactive intestinal polypeptide [82].

## 8. Factors That May Influence Globin Expression and Detection

There are considerable discrepancies between studies regarding the presence, distribution, and regulation of Ngb and Cygb (see above), which may be due to a number of factors. Some methodical aspects and physiological parameters that may be responsible for the discrepancies are briefly discussed below.

A major problem, not unique to globin research, is that many studies have been performed only once. Most reports stand alone in terms of methodical aspects and are therefore not easily comparable. Scientific “gold” standards require that a published result be confirmed at least once (“repetitio mater scientiae est”) in another laboratory and by complementary methods [83].

### 8.1. Technical Parameters That May Influence the Detection of Neuroglobin and Cytoglobin

A wide range of methods have been used to study globin expression. These include blots such as Southern blots to detect specific DNA sequences, Northern blots to detect specific RNA sequences, and Western blots to detect specific proteins using antibodies. In situ hybridization (ISH) is used to localize specific nucleic acid within fixed tissues and cells, providing spatial and temporal data on gene expression. Immunohistochemistry (IHC) uses specific antibodies to localize substances such as proteins under the light microscope or electron microscope (immunocytochemistry). Another methodical approach to identify proteins such as globins is mass spectrometry, an analytical method for characterizing chemical compounds and, among other things, identifying substances in body fluids or organs [84].

It should be emphasized that each of these methods per se does not explain the functions and regulatory mechanisms of substances, but rather adds mosaic pieces to the understanding of proteins such as globins. It is therefore conceivable that differences between detection methods may contribute to the relative variability of the results.

For the study of detailed Ngb and Cygb distribution, IHC has been the most widely used method for the detection, localization, and semi-quantification of antigens in single and grouped cells because of its convenience, reliability, and versatility. In IHC, antigen–antibody complexes are visualized by light microscopy using chromogens or fluorescent markers. The latter has proved particularly useful in multiple labeling studies or in combination with neuronal tracing methods using fluorescent dyes.

#### 8.1.1. Antibodies

The most critical issue is the specificity of the probes and antibodies. In the case of globins, different antibodies have been used for immunohistochemical detection. It is clear that antibodies must be of high quality and specificity. We found that several commercially available antibodies did not meet the requirements and did not give reliable and reproducible results. There is still a lot of discussion about this, but comparative analyses of different antibodies using different fixation methods would be very elaborate and are not yet available.

In our studies, Ngb antibodies directed against a synthetic peptide covering the conserved amino acid positions 55–70 of mammalian Ngb (H_2_N-CLSSPEFLDHIRKVML-CONH_2_) were produced by either Strategic Biosolutions Inc. (San Diego, CA, USA; see [33]) or Eurogentec (Köln, Germany, see [55]). Affinity-purified antibodies were applied to perfusion-fixed brain material sectioned 40 µm on microtome or cryostat. For specificity controls, antibodies were blocked by preabsorption with recombinant Ngb at 5–15 mg recombinant protein per ml. Results were confirmed by a commercially available antibody (Rabbit Anti-Neuroglobin, Sigma-Aldrich Cat# N7162, RRID: AB_796158; Lot 017K4766). It was also blocked by preabsorption and did not produce specific staining in brain sections from Ngb KO mice. These controls were consistent with the requirements for antibody validation summarized by the International Working Group for Antibody Validation [85].

In our studies, Cygb was detected using custom-made, rabbit-raised antibodies directed against amino acid positions 2–16 of the N-terminus (for further details, see [42]). Specificity was tested as described above.

As antibodies are usually used in excess concentrations, it is of utmost importance to down-titrate to the lowest working dilution before preabsorption with different antigen concentrations (see [86] for review). Unfortunately, this is often neglected due to technical and methodical uncertainties. If two or more antigens are detected by immunofluorescence, possible cross-reactivity between primary and secondary antibodies must be excluded. Dye-swap experiments must guarantee that antibody staining patterns are independent of the fluorescent dye.

Immunocytochemistry, i.e., visualization of antigen–antibody complexes by electron microscopy, as well as the interpretation of the results, have been the subject of much debate. Both pre- and post-bedding procedures ultimately failed to live to expectations and were rarely used in neuroscience studies.

#### 8.1.2. Fixation for Immunohistochemistry

Since differences in antibody staining resulting from varying fixation parameters were frequently observed [87], a brief commentary on these parameters is warranted. Immersion in fixative frequently resulted in uneven tissue fixation. Consequently, transcardial perfusion with PLP-solution [88] immediately following the administration of diethyl ether or carbon dioxide overdose to the animals were employed (details are given in refs. [46,68]). This reliable and widely utilized method was proposed as a fixative for light and electron microscopic analysis, providing consistently good preservation of morphology and intense antigen labeling with minimal background. Tissue specimens were post-fixed, cryoprotected with 30% sucrose, and sectioned at 20–40 µm thickness on a cryostat or freezing microtome. Incubations with the aforementioned antibodies were performed in PBS with 1% normal donkey serum and 0.1% Triton-X 100 on a glass slide or, in most cases, free-floating.

Despite the wide variety of detection parameters, many similarities were observed in the staining patterns. They have been analyzed in the preceding sections.

### 8.2. Physiological Parameters That May Influence Globin Expression

A plethora of factors and parameters likely exert an influence on the outcomes of studies, with many studies demonstrating that these results are attributable to physiological variables such as age and sex of the subjects. Conversely, factors such as animal housing conditions or cell line purity, although not overtly apparent, may exhibit considerable variation between laboratories. It is imperative to acknowledge these elements when undertaking data categorization. The ensuing discourse will address select aspects of this multifaceted issue.

#### 8.2.1. Age

The age of the investigated individual is frequently a factor that influences protein synthesis. In the context of globins, there is evidence from several sources to suggest that there is an age-related decline in expression. For instance, Ngb protein expression has been observed to decrease with advancing age (3 to 12 to 24 months) in the mouse brain [89], and has been reported to decline in mice even before 3 months of age [81]. Similar observations have been made in human studies, with Ngb expression levels demonstrating a decline with increasing age [90].

A particular role for Cygb may be present in the inner ear of aged individuals. Initial studies identified Cygb as one of the genes most markedly upregulated in the cochlea of senile rats, and it was found to be most strongly correlated with age-associated hearing disorders [72]. Secondly, it was observed that aged Cygb-KO mice exhibited severe balance impairments and multiple organ abnormalities [74].

#### 8.2.2. Sex

A substantial body of evidence has emerged, demonstrating significant disparities between the structural, physiological, and pathophysiological parameters of female and male organisms. However, while no study has directly addressed the issue of potential sex disparities in globin expression, regulation, or function, certain reports have documented divergent expression patterns between female and male subjects. For instance, higher Ngb immunoreactivity was observed by immunofluorescence in the striatum of female mice at 7 and 13 weeks of age when compared to male animals [81]. Conversely, higher Ngb levels were found by Western blot in the hippocampus of female mice compared to male mice [91].

In healthy humans, Ngb expression was found to be lower in females [90]. In patients suffering from Alzheimer’s disease (AD), Ngb expression was found to be increased in both sexes, suggesting upregulation by the disease process. However, this increase was found to be more pronounced in women than in men, which is consistent with their increased AD risk. In general, when evaluating hormones and related molecules in female mammals of reproductive age, it is necessary to take into account the estrous cycle stage. This is particularly necessary when investigating brain regions related to parameters of reproduction, such as the hypothalamus. The rationale behind the exclusive use of male subjects in experimental designs is not fully elucidated, but it is evident that the findings may not be universally applicable. This point has been frequently overlooked in the literature on globin, and in some studies, the animals’ sex (sometimes incorrectly termed “gender”) has not been specified.

The extant literature does not provide any indication of sex-related differences in the context of Cygb.

#### 8.2.3. Time of Day and Season

It has been demonstrated that the majority of body parameters and functional aspects are subject to the influence of the endogenous circadian clock. In mammals, the suprachiasmatic nucleus, located in the hypothalamus, serves as the primary regulatory center for the circadian rhythm. This rhythm is governed by a complex network of neural and hormonal signals, which include the sympathetic nervous system and the pineal gland (cf. [92]). However, the available literature provides only limited information regarding the potential influences of daytime or season on globin expression. In most studies, the information regarding the timing of tissue harvesting is not provided, hindering the ability to accurately assess the potential influence of diurnal and seasonal variations on globin expression.

Research has identified regular changes in body parameters, with the most prevalent being daily (circadian) rhythms, followed by seasonal [93], monthly (see Section 8.2.2), and even weekly rhythms (triggered by the working rhythm of the animal keepers). Consequently, any study that contemplates these fluctuations must adhere to a stringent day/night light regimen, ensuring an absolute absence of white light during nocturnal periods. However, it is acknowledged that this is not always feasible in animal housing facilities. Based on personal experience, it is evident that strong precautions and controls are necessary to ensure the implementation of absolute light/dark regimens without any light cues during the dark phase.

#### 8.2.4. Stress and Anesthesia

The potential repercussions of stress during housing, rearing, and experimental treatment of animals are of significant interest, given the established correlation between stress response and expression patterns [71]. A considerable degree of variability has been identified in “standardized” animal facility conditions, which often deviate from the desired uniformity, exhibiting significant variation. Recent studies, not related to globins, have identified numerous factors in animal housing that may induce stress. The following section will present some of these factors.

As posited by Mo and colleagues [94], the outcome of rearing may be influenced by factors such as the sex of the animal caretakers. The presence of pheromones from animals housed together, or even from staff members or experimenters, has been demonstrated to trigger these processes [95,96]. Furthermore, discrepancies in housing facilities due to noise may elicit varied responses to experimental conditions. Beyond that, it is self-evident that animals must be held under constant conditions (same light:dark regimen, constant room temperature, standard food and water available ad libitum).

Another significant factor to consider is the type of anesthesia administered during experimental procedures, such as surgical operations. Substances such as ether, ketamine, propofol, or isoflurane have been found to affect brain metabolism [97]. Volatile anesthetics have also been observed to influence expression patterns. Isoflurane, a widely utilized agent in experimental animal studies, has been observed to enhance the permeability of the blood–brain barrier and upregulate hypoxia-inducible factor 1α [98,99]. According to Fordel and coworkers [100], “the mechanism of induction of cytoglobin is regulated by the hypoxia-inducible factor 1, a posttranscriptional regulated transcription factor controlling several hypoxia-inducible genes”. Moreover, agents that modify cerebral blood flow or influence neurotransmission may distort study outcomes in globin research [101,102]. Furthermore, the method of killing may influence Cygb expression, at least at the mRNA level. The impact of external factors, such as olfactory cues and the time interval preceding animal sacrifice, on study outcomes also warrants consideration.

### 8.3. Cell Lines

In recent years, mounting evidence has indicated that cell lines utilized in research may be contaminated or misidentified [103]. The authors reported that a significant number of papers were reliant upon misidentified cells. Several of these cell lines remain in active use, and the affected publications have not been retracted nor accompanied by a note of concern. It is therefore important to consider the possibility that this may also apply to globin research, and that discrepancies in results may be attributable to problems with the cell lines.

### 8.4. Some Notes on Nomenclature and Tissue Composition

It is evident that a recurrent theme in research is the comparison of data from the “cortex” and “cerebellum”. This comparison may be problematic as both cerebrum and cerebellum consist of a cortex (gray matter) covering the medulla (white matter). Given that the term “cortex” refers exclusively to the cerebral cortex devoid of underlying white matter, and that “cerebellum” signifies the entire cerebellum (cortex cerebelli and underlying white matter), a direct comparison between these specimens becomes challenging, as their initial conditions differ. This aspect is of crucial importance as it has the potential to compromise the validity of the data. For instance, if the cerebellar cortex is separated from the medulla, Ngb protein or mRNA expression would be expected to show higher levels compared to a homogenized whole cerebellum.

In the cerebellar cortex, the stratum granulosum consists of numerous small-sized neurons with very little cytoplasm, while the stratum ganglionare is distinguished by a small number of large Ngb-expressing Purkinje neurons with substantial cytoplasm, as well as a preponderance of astrocytes. Conversely, the medulla is characterized by a significant presence of glial cells, predominantly oligodendrocytes that do not express GFAP and thus are not easily identified by IHC. It is important to note that Ngb levels, although high in each of the small number of cerebellar Purkinje cells, will be low in whole cerebellum (and vice versa in the hypothalamus). A comparison of Ngb-mRNA levels in the latter structure with those in other brain regions was made using publicly available transcriptome data [4]. This prompts the question of what this reflects. Could these elevated levels be indicative of increased expression in individual neurons? Or is it merely indicative of increased neuronal volume, attributable to larger neuronal somata and reduced intercellular compounds (neuropil) in the hypothalamus of a given region? It is important to note that this item may be misleading in the discussion of intracellular Ngb levels deduced from homogenized tissues. It is therefore recommended that the magnitude of Ngb expression is considered at the single-cell level rather than at the regional level, particularly when comparing regions with large variation in soma sizes such as the hypothalamus and hippocampus.

It is important to note that a further uncertainty associated with bioinformatic work on publicly available data sets is that researchers are entirely reliant on the information provided, yet they are unaware of the individuals responsible for microdissecting the tissue and the extent of their expertise.

## 9. Mechanisms of Globin Regulation

An intriguing facet in the examination of Ngb and Cygb function pertains to the regulation of their expression under challenging conditions. One such condition is experimentally induced hypoxia, frequently induced by means of ischemia or ischemia/perfusion.

A substantial body of literature has documented the effects of experimental hypoxia in animal models and/or in cell culture. Specifically, studies utilized PC12 cells, a cell line derived from a pheochromocytoma of the rat adrenal medulla, and HN33, an immortalized cell line derived from mouse hippocampal neurons fused with neuroblastoma cells. Some authors also used the ischemia–reperfusion (IR) model generated by transient occlusion of a cerebral artery.

### 9.1. Neuroglobin

The results obtained demonstrate that, in general, the condition of hypoxia results in the upregulation of Ngb expression. However, it should be noted that divergent results were obtained under these conditions, which may be attributable to the varied methodical approaches employed.

In vivo studies in animals have demonstrated that Ngb protein expression is increased by focal ischemia [104], or is slightly upregulated in the “brain” [105]. Ischemia–reperfusion resulted in upregulation of Ngb in the cerebral cortex and downregulation in the hippocampus, while it increased in serum [35]. In the mole-rat Spalax, Ngb levels were found to be elevated under normoxic conditions but downregulated during hypoxia [38].

Conversely, a two-hour hypoxic exposure of animals did not result in alterations in the distribution or intensity of staining in any brain area [75]. Moreover, no alterations were detected in the brains of rats following in vivo ischemia [106].

In cell culture, Ngb-mRNA was enhanced in PC12 cells, and Ngb expression increased in vitro neuronal cells from embryonic rat cerebral cortex [104]. Ngb was upregulated by up to fourfold in HN33 [105], following severe hypoxia for a duration of 24 h [106]. Conversely, no alterations were detected following one or two weeks of 10% O_2_ hypoxia [25].

In addition to the regulatory mechanism involving O_2_ levels, evidence suggests that Ngb expression is influenced by hormones. For instance, Ngb expression in the brain is influenced by thyroid hormone levels, both in states of deficiency and excess [107]. Additionally, 17β-estradiol has been observed to induce Ngb expression through estrogen receptor-α binding to genomic regulatory regions [108]. The mechanisms by which these factors act may involve the translocation of Ngb to mitochondria, thereby facilitating neuronal and glial cell adaptation to injury [109].

For further aspects, the reader is referred to the extensive review by Van Acker and colleagues [3], who compiled many mechanismsegulating Ngb expression on both mRNA and protein levels in vitro and in vivo. For a more detailed discussion of this topic, please refer to Section 12.1 of the present review.

### 9.2. Cytoglobin

As with Ngb, Cygb protein appears to be upregulated in several tissues upon hypoxia, by as much as approximately 5-fold in HN33, suggesting that Cygb is a hypoxia-inducible gene transcriptionally upregulated during hypoxia [100]. The underlying mechanism is believed to involve the induction of HIF1α, which in turn targets nNOS and Cygb [110].

However, contradictory results have been reported in the literature, with some studies demonstrating a decrease in Cygb expression under hypoxic conditions [45], and others reporting no significant changes [47]. Notably, no effects were observed on Cygb expression in the mouse brain following permanent middle cerebral artery occlusion.

In a manner analogous to Ngb, evidence suggests that Cygb expression is influenced by thyroid hormones [107].

In contrast to the findings related to Ngb, the association between Cygb and mitochondria has not been demonstrated, but rather suspected [111].

## 10. Living Without Globin

The investigation of the functional aspects of a substance of interest is facilitated by the utilization of knockout or silencing mouse models. A comparative analysis of specific parameters between wild-type (WT) and knockout (KO) animals is also employed.

### 10.1. Living Without Neuroglobin

To date, the number of studies dealing with Ngb-KO animals is limited [66,70,112,113], with the majority focusing on visual and auditory functions. The effects of Ngb-KO on light-dependent gene expression patterns in the mouse retina revealed no significant differences [112]. However, the study design did not include the testing of crucial visual functions such as acuity or color vision, nor were electroretinograms recorded from the knockout animals. Nevertheless, interesting insights came from a study in which Ngb expression in the retinal ganglion cells of adult rats was reduced by in vivo electroporation of anti-Ngb RNA [114]. This resulted in a reduction in visual performance as measured by optomotor head-tracking tests. The relevance of this rather unphysiological and invasive procedure to real-life situations remains to be seen. However, recent results have demonstrated a suppressed signal associated with hemodynamics and oxidative metabolism in the retinae of Ngb-KO mice [66].

A minimal alteration in light-induced retinal gene expression was observed in Ngb-KO mice, with ATP8A2 being among the genes that showed decreased expression [113]. This ATP-dependent lipid flippase, which translocates aminophospholipids from the exoplasmic to the cytoplasmic leaflets of membranes, is highly expressed in the CNS and retina. Its deficiency has been shown to result in a loss of hearing and vision skills [115]. While the integrity of visual parameters in Ngb-KO remains to be further tested, the apparent functional relation of Ngb and ATP8A2 for hearing is of high interest and warrants further investigations.

In a similar manner, the absence of Ngb gave rise to only minor alterations in the auditory system. As previously mentioned, the present study represents the only one to date in Ngb-KO mice, in which auditory function was assessed by measuring distortion-product otoacoustic emissions and auditory brainstem responses [70]. In summary, the absence of Ngb leads to only marginal impairments in hearing ability, and the recovery of hearing function after acute noise trauma is marginally better in wild-type mice. A moderate but statistically significant decrease in the latest peak-to-peak response amplitude (originating in the IC) was observed in KO mice four weeks after trauma [70]. However, the effects of experimentally induced impairment of hearing response, as measured in the IC, were reverted by in vivo transfer of the Ngb gene in mice [116]. The potential functional role of Ngb in the efferent system, as indicated by Ngb expression in olivocochlear neurons [33], necessitates further investigation.

In addition, there is compelling evidence that oxidative stress, for instance, resulted from acoustic overstimulation, and mitochondrial dysfunction underpins acquired sensorineural hearing loss. The underlying mechanism is hypothesized to involve the generation of ROS, which then constricts the cochlear vasculature, leading to reduced cochlear blood flow (cf. [117]). The involvement of Ngb and Cygb in these processes is a hypothesis that merits further investigation.

The pervasive distribution of this globin family member within the brain, particularly within the auditory system, stands in clear contrast to the dearth of knowledge surrounding its function. The results of this study suggest that the loss of Ngb does not affect neuronal viability during hypoxia in vivo. Instead, Ngb deficiency appears to enhance the hypoxia-dependent response of HIF1α and c-FOS protein, while also altering the transcriptional regulation of the glycolytic pathway. Bioinformatic analysis of differential gene expression has yielded novel predictions suggesting that chromatin remodeling and mRNA metabolism are among the key regulatory mechanisms that occur during adaptation to prolonged hypoxia [112].

### 10.2. Living Without Cytoglobin

To date, the number of studies addressing neurons in Cygb-KO mice remains limited, with only a few having been published to date [118,119,120].

Research conducted using mouse KO models has suggested a role for Cygb in regulating vascular tone through NO deoxygenation (scavenging) [119] and in protecting cells from superoxide toxicity via its superoxide dismutase function [120].

It is interesting to note that only minor changes were observed in the transcriptomes of selected brain regions of Cygb-KO mice [118]. The potential implications of this observation, specifically whether it is attributable to the expression of residual Cygb-mRNA in this particular KO model, remain to be elucidated [118]. A masking effect of Cygb-negative cells may also be crucial, given that pyramidal neurons of the hippocampus, although exhibiting strong Cygb immunofluorescence, make up only a very small proportion of hippocampal volume [46].

## 11. Living on More Globin

In an attempt to elucidate the functions of globins, transgenic animal models were constructed, expressing levels of neuroglobin and cytoglobin that exceeded the physiological levels found in the original species.

### 11.1. Neuroglobin Overexpression

The functional aspects of overexpression (OE) have been shown to be effective in counteracting the effects of experimentally induced brain lesions and/or downregulation of Ngb. For instance, the volume of cerebral infarcts subsequent to the occlusion of the middle cerebral artery was diminished in transgenic animals [121], and Ngb OE reduced brain lesion size and sensorimotor deficits following experimentally induced traumatic brain injury in mice [122].

The constitutive OE of Ngb was found to be confined to a limited number of brain regions, including cortical areas, the hippocampus, the caudate putamen, and the cerebellum [123]. In these regions, Ngb appeared to counteract the decrease in Ngb that occurs with age, thereby mitigating age-induced neuromotor dysfunction [124]. However, a subsequent study failed to demonstrate enhanced recovery of sensorimotor deficits [125]. Moreover, the administration of Ngb into SH-SY5Y (human neuroblastoma cells) and RGL-5 (retinal ganglion cells) cells did not augment cell viability following oxygen deprivation in vitro [126].

The mechanisms of protective function of Ngb OE may include the preservation of mitochondrial function and mitigation of nitric oxide-mediated injury in PC12 cells and primary cortical neurons in vitro [127,128]. Ngb has been shown to inhibit mitochondrial pathways of apoptosis by binding to Cytc, as found in Ngb-overexpressing SH-SY5Y cells [129]. Very recently, a study using mass spectrometry showed that NGB OE acts as a positive regulator of autophagy in a human neuroblastoma cell line [84]. Furthermore, it has been demonstrated that Ngb enhances the expression of endothelial NOS in vascular endothelial cells, thereby providing vasomotor protective mechanisms [121]. In transgenic Ngb OE mice exposed to IR, hippocampal ROS production was reduced compared to control animals, suggesting that Ngb protects through its antioxidant properties [130].

To date, no studies have been conducted to examine auditory functions in Ngb OE models. Given the loss of function observed in Ngb-KO mice [70], which mirrors the age-related decline in hearing parameters [131], it would be intriguing to ascertain whether the age-related decline in Ngb synthesis [89] contributes to the parallel functional decline of hearing ability [131], and whether Ngb OE has the capacity to counteract this phenomenon.

### 11.2. Cygb Overexpression

Most studies investigating the effects of Cygb OE have focused on cancer and other diseases linked to collagen synthesis, such as fibrosis. For instance, it has been demonstrated that Cygb OE protects the liver against damage-induced fibrosis [132], likely by reducing collagen synthesis [133].

A paucity of studies has been conducted on the effects of Cygb OE on neuronal cells or cell lines. The majority of these reports investigated the protection of SH-SY5Y neuroblastoma cells against oxidative stress-induced cell death. For instance, in experiments involving induced hypoxia, Cygb and HIF-1α expression levels increased in SH-SY5Y cells, while the incidence of both apoptotic and necrotic death was reduced when the cells were transiently transfected with plasmid DNA containing the Ngb or Cygb sequence [134]. These results demonstrate a strong relationship between HIF-1α and Cygb during hypoxic injury when both factors are upregulated [135]. It was concluded that Cygb (or Ngb) act as ROS scavengers under ischemic conditions such as oxygen and glucose deprivation [136].

A study into the relationship between Cygb OE and Ngb expression, including the impact observed in knockout models, would be a worthwhile endeavor.

## 12. What Are the Functions of Neuronal Globins in Mammals?

As indicated previously, the studies on KO, silencing, and OE contributed to the advancement of knowledge concerning the functions of Ngb and Cygb in neurons and neuronal networks.

### 12.1. Neuroglobin

The initial hypothesis proposed that Ngb functions as a protein responsible for the storage and transportation of oxygen [1]. However, the relatively low concentration of Ngb in the brain (see below for discussion)—with the exception of the retina—appears to contradict this assumption. Instead, the hypothesis is that Ngb functions as a scavenger of NO and reactive oxygen species. Ngb may also function as a sensor that detects low oxygen levels and initiates signal transduction to protect cells against hypoxic conditions (cf. [11]).

The protective effects of Ngb against hypoxic/ischemic conditions in an animal model of middle cerebral artery occlusion have indeed been demonstrated. Ngb application reduced infarct size, while its blockade by intraventricular Ngb antisense infusion exacerbated the effects [137]. Ngb OE was associated with a decrease in mitochondrial DNA damage following retinal ischemia–reperfusion [138]. The neuroprotective function of Ngb is believed to be a result of its inhibition of apoptosis pathways, related to Cytc released from mitochondria to reset the trigger level for the onset of apoptotic events [5,139]. The hypothesis is that Ngb migrates with mitochondria to the source of oxygen in situations of insufficient oxygen supply [140].

There is also evidence to suggest a role of Ngb in neurite outgrowth, since this globin was found to be upregulated in neurites of primary cultured cerebral cortical neurons, where it was predominantly found in cell processes. In mouse neuroblastoma N2a cells, silencing Ngb inhibited neurite outgrowth, while OE of Ngb promoted axonal outgrowth of cortical neurons in vitro [141]. In the adult neurogenic subventricular zone, Ngb expression has been observed in cells identified with the neuronal lineage marker doublecortin [142].

As demonstrated in Figure 4E,F, immunofluorescence studies conducted in our laboratory reveal the presence and partial co-expression of Ngb and nNOS in pericytes. These cells play an eminent role in the context of the neurovascular unit mechanism, i.e., they are part of a network consisting of neuronal, glial, and vascular cells and can develop into these cell types after challenging conditions such as hypoxia and ischemia (cf. [143,144]). In particular, those cells that express Ngb have been shown to exhibit reduced leakage in the blood–brain barrier after experimentally induced ischemia [145].

A comprehensive review of studies examining the protective effects of Ngb was provided by Dietz [146]). The majority of studies concur on the upregulation of Ngb in brain tissue, both in vitro and in vivo, but a definitive regulatory line is absent, given the findings that hypoxia and ischemia may result in either upregulation or no effect on Ngb expression. These discrepancies may, however, be attributable to variations in experimental conditions and the specific brain regions examined. Furthermore, numerous inquiries concerning its mechanism persist. For instance, the question of whether Ngb operates solely within the cell or if it possesses an extracellular site of action remains unanswered. The potential for its release from neurons, its eventual role as a transmitter or modulator, and the possible existence of a receptor structure for Ngb remain to be elucidated. Moreover, the mechanisms of active transportation within the axon and of passive diffusion from the cytoplasm into the plasma of cell processes have yet to be investigated.

Furthermore, it is imperative to determine the half-life of Ngb, and whether the molecule undergoes dissipation upon action and, if so, whether it is subsequently re-accumulated. In addition, the temporal progression of mRNA and protein expression in response to challenges is to be examined. Finally, the relationship between high intracellular protein levels and high demand or low consumption, as well as the relationship between soma size and mRNA expression levels require further investigation.

### 12.2. Cytoglobin

The study of Cygb function is a multifaceted endeavor, given the intricate nature of the subject. The observation of Cygb immunoreaction in the cytoplasm and the nucleus of neurons but not in cells of the fibroblast lineage may indicate differential functions of the protein between neurons and, for example, fibroblasts. It is noteworthy that this specific location was observed exclusively for Cygb and not for other globins such as neuroglobin.

The physiological role of Cygb in neurons remains to be elucidated [6]. A significant proportion of the extant knowledge regarding the molecular function of Cygb is derived from studies employing animal models, wherein KO, silencing, or OE techniques have been utilized (see [7]). However, the effects of these manipulations have been investigated in several studies on organs such as the kidney or liver [6], while the possible effects on brain or neuronal parameters remain enigmatic. Only a limited number of reports have addressed the topic of abolished or attenuated expression of Cygb on structural or functional parameters of neurons or neuronal networks [118,119].

In addition to its functions as a tumor suppressor gene in non-neuronal cells [147], an important physiological function of Cygb appears to be related to collagen synthesis. Fibroblasts and analogous cell types have been shown to synthesize collagen in the presence of elevated levels of oxygen consumption. Consequently, Cygb may function as an O_2_ supplier to prolyl 4-hydroxylase, thereby facilitating the hydroxylation of proline residues to procollagen.

In view of the higher concentration of Cygb in active fibroblasts and related cells in comparison to inactive specimens that have ceased collagen synthesis [42], a clear relationship between Cygb and collagen synthesis in these cells is evident. Furthermore, Cygb expression and collagen synthesis increased in parallel under conditions of hypoxia [42,45]. While the production of collagen is not inherently associated with neurons, an analogous function in these cells may be the synthesis of transmembrane collagens that have been observed in the formation and function of neural circuits [148,149].

The presence of Cygb in both the neuronal cytoplasm and nucleus suggests the existence of an additional specific feature of Cygb in neuronal tissue, possibly a sensing function, e.g., of O_2_ or NO, thus modulating adaptation processes of neurons [44] (see Section 2.2 for more details on the nuclear location of Cygb). Furthermore, the potential role of Cygb in NO metabolism remains probable, given its relatively high degree of colocation with NO. For instance, in the mouse auditory brainstem, the distributions of Cygb and nNOS revealed that the percentage of Cygb neurons that were also nNOS-immunoreactive averaged at 74% of the total Cygb cell number [68]. This relatively high proportion of neurons expressing both molecules suggests that they interact in functions such as delivering oxygen during NO production or scavenging excess nitric oxide. Earlier reports have documented individual findings of co-expression in specific brain regions [27,38,46,150]. Cygb also possesses dioxygenase activity, which involves the conversion of NO to nitrate. This process renders NO harmless at high concentrations, despite its potential to damage cells [151]. The concomitant presence of NO and Cygb suggests the potential involvement of Cygb in NO metabolism (cf. [9]).

In the context of the auditory system as a neuronal model system, the potential for further mechanisms by which cytoglobin may influence functions is evident. Given that Cygb functions as an oxygen-sensing molecule, it may play a role in regulating cochlear microcirculation. It is transported into the cochlea by olivocochlear neurons (see Section 5.2), but a more likely scenario is that cochlear Cygb stems from pericytes. These cells play a pivotal role in regulating the microcirculation of the cochlea, thereby contributing to the reduction in blood flow following ischemia [152]. This process is likely to involve a decrease in capillary diameter (see [153] for further details). However, Cygb expression would be augmented in response to local hypoxia mediated by the hypoxia-inducible factor (HIF)-1α. This may result in increased vascular endothelial growth factor (VEGF), the relaxation of capillary pericytes, and the subsequent increase in microvessel diameter [8,154], thus improving blood supply of the organ of Corti.

In addition to the analysis of projection patterns and neurochemistry, further research is necessary to determine the functional role and potential regulatory mechanisms of Cygb-expressing neurons. Recently, Cygb was identified as a factor in synaptic plasticity induced by tetrodotoxin, a sodium channel inhibitor that suppresses network activity [155].

## 13. Conclusions

In conclusion, a wide spectrum of publications using various methodical approaches reported the distribution and regulation and sought to decrypt the functions of neuroglobin and cytoglobin in mammalian nervous systems and in interacting structures such as the sensory organs (cf. [6,8,15]). Ngb expression in neurons is transiently increased upon hypoxia and provides protective effects in response to hypoxia, ischemia, and oxidative stress. Thus, NGB may serve as a promising candidate for the treatment of neurological diseases [156]. Furthermore, Ngb expression has been observed in astrocytes under specific physiological or pathological conditions. Cytoglobin has the capacity to bind O_2_, NO, and CO, and its neural effects include the association with cytoprotection against oxidative stress through HIF regulation, as well as acting as a NO dioxygenase. It is also involved in collagen synthesis and probably exerts important functions in the regulation of microvascular blood supply by its NO-mediated regulation of pericyte diameter. The presence of Cygb in the cytoplasm and nucleus is a unique feature within the globin family, but its significance is still unclear. As patterns in neurons or neuronal assemblies expressing either globin are not recognizable, it is difficult to ascertain a correlation between globin-expressing neurons and their functional significance. The necessity for further research into globins is evident, with the aim of answering numerous unresolved questions, some of which are addressed in this paper. Further research is required to elucidate the function of neural, endocrine, and sensory organs in knockout and overexpressing animals, including the sensory functions of knockout animals. Furthermore, the possible effects of experimental blocking of axonal transport are of interest. Additionally, further exploration is necessary to elucidate the multifaceted interactions between neuronal and glial cells with respect to globin expression within the nervous systems.

## Abbreviations

CB—carotid body, CNS—central nervous system, Cygb—cytoglobin, Cytc—cytochrome c, HIF—hypoxia-inducible factor, IHC—immunohistochemistry, ISH—in situ hybridization, KO—knockout, Ngb—neuroglobin, nNOS—neuronal nitric oxide synthase, NO—nitric oxide, OCNs—olivocochlear neurons, OE—overexpression, PNS—peripheral nervous system, mRNA, RT-PCR, SGN—spiral ganglia neurons.
